# Do smartphone applications in healthcare require a governance and legal framework? It depends on the application!

**DOI:** 10.1186/1741-7015-12-29

**Published:** 2014-02-14

**Authors:** Esmita Charani, Enrique Castro-Sánchez, Luke SP Moore, Alison Holmes

**Affiliations:** 1 Centre for Infection Prevention and Management, Imperial College London, Du Cane Road, London W12 0NN, USA

**Keywords:** Apps, Ehealth, Mhealth, Smartphone, Technology adoption

## Abstract

The fast pace of technological improvement and the rapid development and adoption of healthcare applications present crucial challenges for clinicians, users and policy makers. Some of the most pressing dilemmas include the need to ensure the safety of applications and establish their cost-effectiveness while engaging patients and users to optimize their integration into health decision-making. Healthcare organizations need to consider the risk of fragmenting clinical practice within the organization as a result of too many apps being developed or used, as well as mechanisms for app integration into the wider electronic health records through development of governance framework for their use. The impact of app use on the interactions between clinicians and patients needs to be explored, together with the skills required for both groups to benefit from the use of apps. Although healthcare and academic institutions should support the improvements offered by technological advances, they must strive to do so within robust governance frameworks, after sound evaluation of clinical outcomes and examination of potential unintended consequences.

## Background

The use of smartphone and tablet applications (apps) within healthcare is rapidly expanding [1-6] and now appears socially and professionally acceptable [7]. The Food and Drug Administration has recently published guidelines defining the group of apps to which it intends to apply its authority [8], but whether US legislation should follow continues to be fiercely debated [9,10]. The European Commission has also issued legislation regulating the development and use of medical devices, which covers software utilized in healthcare apps [11]. Many view these steps as a positive move towards recognition of the role of apps in healthcare as decision support tools; others, however, may see this additional governance as cumbersome and a hindrance to effective use of this rapidly evolving and changing technology. In the context of the clinical quality improvement agenda, the question is whether the use of apps needs to be considered in the same light as other healthcare interventions and subject to a similar degree of pre- and post-implementation evaluation and economic assessment. This is of particular importance for apps designed to have a direct impact on clinical decision-making, including medication dose calculation, adjustment of therapy, and for diagnostic purposes [12,13]. Evidence supports the rapid adoption of apps for the delivery of healthcare information and decision support tools to healthcare professionals [14,15] and patients [16], but studies on the impact of apps on patient outcomes are lacking.

The unintended consequences of app use in healthcare remain largely unexplored. The inexorable development of digital technologies moves at a fast pace and current devices will be superseded, as suggested by the advent of wearable devices such as Google Glass and smartwatches [17]. Likewise, some manufacturers and standards will disappear, to be replaced by others. It is pertinent, therefore, to consider the lifespan and sustainability of apps. This is of particular importance when the funding for developing apps for healthcare organizations may not only come from private industry but also from the public sector.

## The need for relevant information governance

The lack of seamless access to patient information across the care pathway remains one of the most pressing issues in healthcare [18]. The absence of an integrated system poses a threat to care quality and patient safety as important clinical decisions have to be made after referring to multiple electronic systems, raising further issues in terms of governance and handling of confidential data. It is possible that the enthusiasm among healthcare professionals to use smartphones to help make clinical decisions stems in part from a frustration with existing information technology systems provided by healthcare organizations. In this context, apps may have provided a ‘quick fix’ for circumventing the underlying problems related to healthcare information technology architecture, rather than being horizontally integrated into the wider health informatics landscape. This in turn is contributing to the observed fragmentation of apps [19].

Apps may provide a robust mechanism for improving out-of-hours care where one reason for poorer patient outcomes may be lack of remote access to data to help inform decision-making by non-resident practitioners. Utilizing apps to overcome this, however, is mired with difficulties, chief among them being access to patient data and patient confidentiality. Existing electronic systems and servers in healthcare operate behind secure firewalls. Providing access to patient data through mobile devices would require careful consideration of the ethical, security and governance aspects of data handling. But this need not be a deterrent. If enacted correctly and systematically, access to patient information through mobile devices can be much more secure than access through paper records, which can be transported and/or photocopied and misplaced much more easily. Data encryption methods specifically designed for mHealth apps are now coming to fruition [18]. However, electronic and digital systems pose their own threats and resilience to malware and cyber attacks must be developed [20,21].

One solution to safe access to patient-level data through mobile devices is for organizations to have clear security and governance rules in place. These may include the provision of devices or registering of all mobile devices used within the organization; registration of individual users; the use of virtual secure networks; and utilization of apps designed to prevent data being stored locally on the device. For those apps that provide patient-level decision support, mechanisms to maintain a decision-making audit trail must be developed. This may entail digital logs or print-out-and-sign signatures of healthcare professionals that should be retrievable and shareable amongst the various apps and electronic and paper systems used to document the care provided for individual patients. Furthermore, such systems may also facilitate sharing of information across patient pathways to support an integrated care approach across primary and secondary care.

## Will increased governance stifle innovation?

The way that healthcare professionals learn and practice is rapidly changing with technological evolution [22], with a resulting increased demand for technology among the workforce [1,2,14,15]. Technological advances cost money and require additional expertise for successful adoption and implementation. It is important to assess existing information technology capabilities within healthcare organizations and their ability to deliver products to meet demand. The digital skill of healthcare professionals is always ahead of the technology that healthcare organizations adopt. While most healthcare professionals are now adept at using smartphones and tablets, they continue to work in healthcare systems that are struggling to renovate their technologies. We are still some way away from the goal of working in a paperless system in healthcare and the opportunity cost of investing in smartphone technology and apps, especially in light of the need for governance, may act as a deterrent. Where healthcare information technology systems are designed with decision support architecture in place (such as electronic prescribing), any additional benefit offered by apps from their point-of-care nature should be weighed against incremental costs. To this end, the Food and Drug Administration has classified applications used in healthcare into those that provide medical and scientific information to supplement expertise such as policy and guidance [14], and those that have diagnostic and intervention potential [8], a decision that has been welcomed. This way the need for legislation and governance can be targeted to certain applications without stifling the use of such technology in healthcare altogether.

Healthcare professionals are not the sole users of mobile technology, as patients and service users also have access to the same tools and potentially to the same apps. Patients need to be at the center of the technological advances in healthcare, and scaling up of technological innovations must include this perspective and consider supporting patient choice of innovation. The impact of healthcare apps upon patient participation and their perception of the clinical experience should not be underestimated [23]. Additionally, there may be other unintended consequences, including changes to patient health-seeking behaviors as a result of access to information through apps.

## Summary

The inevitable future for healthcare is one where electronic and mobile systems play pivotal roles in healthcare delivery. From electronic prescribing systems to dispensing robots and robotic surgery, mobile health solutions are only the latest in a long line of technological innovations. Healthcare and academic institutions should support the use of technology and not stifle technological progress, but the drive for development of apps needs to be supported by robust governance frameworks, and evaluation of the clinical outcomes and potential unintended consequences. A classification system for healthcare applications should be developed that recognizes and delineates the difference between apps that support decision-making, and those which purport to intervene in clinical decisions.

## Competing interests

EC and AH have designed and implemented a smartphone application for the Treatment of Infection Policy at Imperial College Healthcare NHS Trust. This application had no commercial incentive and is available free of charge to staff working at the organization. EC, ECS, LSPM and AH are involved in ongoing research evaluating the use of mobile technology in healthcare.

## Authors’ contributions

EC wrote the first draft of the manuscript. ECS, LSPM and AH contributed to the writing of the manuscript. EC is the nominated guarantor of this article. All authors read and approved the final manuscript.

## Authors’ information

EC is an infectious disease pharmacist currently working in academic research. ECS is an academic nurse undertaking research into health literacy. LSPM is a medical doctor in the field of infection, recently awarded an National Institute for Health Imperial Biomedical Research Centre Clinical Fellowship in the field of antimicrobial resistance and stewardship. AH is a Professor of Infectious Diseases, Lead for Hospital Epidemiology and Infection Prevention and Control at Imperial College Healthcare NHS Trust, and a Co-Director of the Centre for Infection Prevention and Management at Imperial College London. EC, ECS and LSPM are all involved in the development, implementation and assessment of healthcare apps across a multisite academic healthcare institution in London. Furthermore, the team under the leadership of AH is currently exploring the utility of using mobile apps and games accessed on smartphones and tablets to change healthcare professional behaviors in antimicrobial prescribing and infection prevention and control.
